# Feeding Infants: Choice-Specific Considerations, Parental Obligation, and Pragmatic Satisficing

**DOI:** 10.1007/s10677-023-10400-5

**Published:** 2023-06-15

**Authors:** Clare Marie Moriarty, Ben Davies

**Affiliations:** 1 Philosophy Department, Trinity College Dublin, Dublin, Eire Ireland; 2 Uehiro Centre for Practical Ethics, University of Oxford, Oxford, OX1 1PT UK

**Keywords:** Breastfeeding, Formula feeding, Parental obligation, Satisficing, Public health

## Abstract

Health institutions recommend that young infants be exclusively breastfed on demand, and it is widely held that parents who can breastfeed have an obligation to do so. This has been challenged in recent philosophical work, especially by Fiona Woollard. Woollard’s work critically engages with two distinct views of parental obligation that might ground such an obligation—based on maximal benefit and avoidance of significant harm—to reject an obligation to breastfeed. While agreeing with Woollard’s substantive conclusion, this paper (drawing on philosophical discussion of the ‘right to rear’) argues that there are several more moderate views of parental obligation which might also be thought to ground parental obligation. We first show that an obligation to breastfeed might result not from a general obligation to maximally benefit one’s child, but from what we call ‘choice-specific’ obligations to maximise benefit within particular activities. We then develop this idea through two views of parental obligation—the Dual Interest view, and the Best Custodian view—to ground an obligation to exclusively breastfeed on demand, before showing how both these more moderate views fail. Finally, we argue that not only is there no general obligation to breastfeed children, but that it is often morally right not to do so. Since much advice from health institutions on this issue implies that exclusive breastfeeding on demand is the best option for all families, our argument drives the feeding debate forward by showing that this advice often misrepresents parents’ moral obligations in potentially harmful ways.

## Introduction

We don’t need to look hard for examples of new mothers being told they ought to solely breastfeed their young children. The World Health Organization (WHO 2022a) recommends breastmilk as “the ideal food for infants”, laments that “nearly 2 out of 3 infants are not exclusively breastfed for the recommended 6 months”, and reports that “[b]reastfed children perform better on intelligence tests, are less likely to be overweight or obese and less prone to diabetes later in life.” WHO and UNICEF recommend that: (1) “children (…) be exclusively breastfed for the first 6 months of life – meaning no other foods or liquids are provided, including water”; (2) “Infants should be breastfed on demand – that is as often as the child wants, day and night. No bottles, teats or pacifiers should be used”; and (3) “From the age of 6 months, children should begin eating safe and adequate complementary foods while continuing to breastfeed for up to 2 years and beyond.” (WHO 2022b). A recent Lancet special issue on breastfeeding contains papers noting in apparent concern that “the consumption of commercial milk formula has been normalised” (Doherty et al. 2023: 415); describing breastfeeding as “a crucial part of the nurturing of infants” (Pérez-Escamilla et al. 2023: 474); recommending a redirection of funds supporting formula feeding “towards maternity care and breastfeeding support services” (Baker et al. 2023: 517); and even proposing that “marketing of [commercial formula milk] products should not be permitted” (Rollins et al. 2023: 487).

Between exclusive breastfeeding and exclusive formula-feeding lie numerous combinations of breastmilk, formula-milk, donor breastmilk, and intravenous feeding. “Breast is best” advocates have varying approaches to non-exclusive options. Advocacy and education organisation, La Leche League International (LLLI), has “Mothering through breastfeeding is the most natural and effective way of understanding and satisfying the needs of the baby” and “For the healthy, full-term baby, human milk is the only food necessary until the baby shows signs of readiness for complementary foods (…)” as two cornerstone statements of their ‘Philosophy’ (LLLI 2022b), suggesting that exclusive breastfeeding is preferable. “Mixing milk” is discussed in “if necessary” terms, and they cite a WHO hierarchy of breastfeeding supplements which places breastfeeding at the top, and formula at the bottom (La Leche League International 2022a).

This paper focuses on two feeding options. We first consider arguments for an obligation to conform with the above guidance, which we call ‘exclusive breastfeeding on demand’ (EBFoD). This obligation contrasts EBFoD with all other feeding options, including ‘mixed’ approaches. We reject this obligation; however, this is consistent with believing in an obligation to *sometimes* breastfeed, or to exclusively breastfeed but at regular intervals rather than on demand; in other words, an obligation to avoid ‘Exclusive Formula-Feeding’ (EFF). EFF is the feeding approach farthest from most official guidance. We argue that EFF is not only reasonable and morally acceptable but is often the morally superior option.

‘Breast is best’ rhetoric is increasingly criticised. A widely accepted worry is that this framing risks harming those who cannot breastfeed or find doing so very costly. A non-exhaustive list includes many adoptive and fostering parents, surrogate parents, some trans and non-binary parents, cis natal mothers with latching issues, single cis men, and couples comprising cis men. Even if the language around breastfeeding allows for excuses (‘breastfeeding is best, but if you cannot breastfeed then bottle feeding is OK’), those who don’t breastfeed may feel they have failed their child (e.g. American Institute for Cancer Research 2022).

Breastfeeding recommendations typically target ‘mothers’, generally assumed to be cis women.^1^ Certainly, cis women comprise the largest group for whom such recommendations are relevant. However, the moral implications have consequences for all who could breastfeed—including some trans fathers and non-binary parents—alongside parents who cannot breastfeed. While we discuss advice that is, in fact, directed to ‘mothers’, our discussion refers to ‘breastfeeding-eligible parents’.

While breastfeeding promotion standardly allows for cost-based ‘excuses’, it is harsher on those who choose not to breastfeed (or even EBFoD) for other reasons. That mothers can be excused from the obligation to breastfeed by *significant* costs implies that lesser costs are insufficient (see Woollard’s (2018a: 134) discussion of Scott (2002)). Precisely how to draw this line is debatable but proponents of EBFoD will all agree that while some reasons for not EBFoD are genuine excuses, others aren’t. Even those who reject an obligation to EBFoD typically focus on inability and significant cost.

The obligation to breastfeed has received considerable philosophical analysis recently, most prominently by Woollard individually ([Bibr CR48][Bibr CR49][Bibr CR50][Bibr CR51]) and with Porter (2017), adding considerable theoretical sophistication to moral dimensions of breastfeeding. Woollard’s central argument suggests that *no* mother has a duty to breastfeed. We agree, but note that Woollard’s discussion focuses on two extreme views of parental obligation. On one hand, Woollard suggests that widespread views about an obligation to breastfeed assume a ‘defeasible duty’ (see below) to maximally benefit one’s child, an extremely demanding view of parental obligation. Alternatively, Woollard considers the view that failure to breastfeed constitutes a significant harm to the child, consistent with a fairly undemanding view of parental obligation.

This leaves unexplored the implications of more moderate views. After outlining Woollard’s analysis of the ‘Maximal Benefit’ view of parental obligation in Section 2, we suggest a less demanding (and thus theoretically stronger) ground for a duty to breastfeed in Section 3.1: unlike Woollard’s target view, which assumes a maximal duty to benefit in all respects, the view we consider suggests that within particular zones of activity there is an obligation to maximally benefit children unless doing so would accrue some level of further cost. In Sections 3.2 and 3.3, we introduce an ongoing philosophical debate on the ‘right to rear’, foregrounding two moderate approaches to parental obligation. These views are the Dual-Interest view—parents may balance obligations to their children against their own interests; and Best Custodian view—parents must benefit their children not *maximally*, but at least as much as a realistic alternative caregiver. We show that neither moderate view supports a universal obligation to EBFoD. Section 4 finishes by considering the implications of our argument for the attitudes that parents, other individuals, and relevant institutions should take towards parents’ feeding decisions. We suggest that the complexity involved in determining which feeding option is best in individual cases means that it is rarely appropriate for institutions or the state to strongly promote one option over others, particularly through selective financial, institutional and other support. While it is questionable whether it would be appropriate for the state to do this *even if* parents have an obligation to breastfeed, the fact that parents do not have such an obligation further strengthens the case that the state should support a range of feeding practices. We also suggest that, given the complexity of the decision, parents are entitled to take a pragmatic satisficing approach to feeding, adopting any of several ‘good enough’ practices without worrying about whether their choice is all-things-considered best.

## Maximal Obligation and Harm Avoidance

Woollard (2018a) suggests that prevailing attitudes imply a ‘defeasible duty’ to maximally benefit one’s children. A defeasible duty is an obligation that can be outweighed by sufficient countervailing reasons. If the duty is *not* outweighed and not performed, blame is appropriate: the agent should blame themselves, and others with appropriate standing, in appropriate conditions, are entitled, even obligated, to blame them. Woollard suggests the best explanation of the rhetoric around infant feeding is that people believe mothers have a defeasible duty to breastfeed. This is, she suggests, grounded in a broader defeasible duty to “perform each action that could benefit” one’s child (2018a: 131), as evidenced by significant restrictions placed on the behaviour of potential mothers, particularly diet and physical activity, on grounds of potential impact on their (future) child.^2^

Woollard (2018a) suggests two arguments for this view, each of which is shown to be flawed. The first conflates having a moral *reason* to do something and having a defeasible duty to do it. There are many things it would be morally good to do, but which are supererogatory, not morally obligatory (though, see Callahan (2019b)).

The second argument moves from the uncontroversial claim that mothers have defeasible duties to benefit their children to the claim that a defeasible duty to benefit someone implies a more specific defeasible duty to perform *any* given action that would benefit them. As Woollard notes (2018a: 139), this is implausible. That mothers have a defeasible duty to benefit their children is a “general principle”, and some general principles allow for discretion in how they are fulfilled: they are ‘imperfect duties’, in contrast to the perfect duties that must always be fulfilled (Callahan (2019a)). For instance, Kant (1785) proposes a perfect duty never to make promises we don’t intend to keep, but merely an imperfect duty to promote others’ happiness; the latter permits flexibility and discretion, the former does not. Woollard proposes a related distinction between *maximal* duties, which must be fulfilled “to the greatest extent possible”, and *non-maximal* duties, which needn’t be. That mothers have a defeasible duty to breastfeed follows straightforwardly from a broader duty to benefit children only if that broader duty is maximal and breastfeeding is the most beneficial feeding option.

Finally, Woollard argues that a maximal duty of beneficence to one’s children would be too costly; since there are innumerable ways mothers could benefit their children, a maximal duty would imply that they are always liable to criticism *unless* they could produce a suitable countervailing consideration as an excuse. This phenomenon of being *constantly* liable would have unacceptable implications, says Woollard, for self-ownership and well-being. Since these costs speak not against specific instances of a putative duty to maximally benefit one’s children, but against that duty in general, Woollard concludes that no such duty exists.

Woollard’s primary case against a duty to breastfeed thus targets a justification resting on a maximal duty to benefit one’s children. Elsewhere Woollard (2018b) considers an alternative view, that failure to breastfeed constitutes a harm. While she doesn’t object as forcefully to this view as to the Maximal Benefit approach, she notes that there are considerable complexities in applying standard analyses of harm to infant feeding.

Thus, in her extant work, Woollard considers two possible grounds of an obligation to breastfeed, sitting at opposite ends of a spectrum: a duty to *maximally benefit* one’s child, and a duty not to (significantly) harm one’s child. We consider more moderate grounding for the obligation to EBFoD before explaining why this too fails. Woollard’s arguments, while powerful, leave open the possibility of an EBFoD obligation grounded in more philosophically popular views of parental obligation; showing that such arguments also fail thus represents an important expansion of her work.

## Choice-Specific Duties

We have noted that Woollard persuasively argues against a duty to breastfeed. The central argument on which she focuses is one which assumes a maximal duty to benefit one’s child. However, one might reject a *general* maximal duty to benefit one’s child, yet think that within *some* types of activity, including feeding, parents should do what is best for their child. If EBFoD is the best feeding method, then even this weaker assumption grounds an obligation to EBFoD. Thus, in this section we outline how such an argument, grounded in a more moderate view of parental obligation, might work. We then show that it too fails.

Consider the distinction between *whether* to undertake some activity, and *how* to do so. Focusing on charitable giving, Pummer (2016) argues that we often have deontological moral options not to benefit others if doing so would be very costly. However, once we decide to take on those costs, we must maximise the good we do where this doesn’t (substantially) increase the cost. Assume you are choosing between keeping £1000, giving it to charity A, or giving it to less effective charity B. You have the moral option to keep the money due to the cost of donating. However, *if* you donate, the choice between A and B involves the same cost, and so you lack further grounds not to maximise the amount of good done. You should, Pummer says, *avoid gratuitous worseness.*

Pummer’s view could clearly apply to parenting decisions. This would imply that for some decisions, if you take on the associated costs, then you should maximise the associated benefit to your children. For feeding, this would imply an obligation to breastfeed—assuming it is the most beneficial feeding method—if one can do so at no extra cost. However, Pummer notes that if we have any reason at all to prefer the less effective charity B, there will be *some* additional cost to instead giving the money to A. He thus considers various stronger versions of his view, where giving to charity A is required if it is slightly, moderately, or even much costlier than giving to B. Thus, a proponent of a duty to breastfeed might hold that when it comes to feeding, slightly, moderately or even significantly greater costs to you cannot justify deciding not to EBFoD.

One issue with this view—as applied to infant feeding, not Pummer’s original version—is that it implies that all parental activities are optional. But even those who reject a duty to EBFoD will not claim that feeding one’s child is optional. We can amend the view to acknowledge this: you have a defeasible duty to maximally benefit your child when undertaking optional activities you choose to do, *and* morally non-optional activities. The duty’s defeasibility depends on which version (slight, moderate, significant costs) of Pummer’s approach we take. But on no version is there a *maximal* duty to benefit children in all possible ways, at all times. Thus, parents have some—maybe considerable—control over whether they take on additional beneficial activities, and have cost-based excuses not to maximally benefit their children within non-optional activities.

Each version is less demanding than the maximal duty that Woollard considers. But each could ground a defeasible duty to EBFoD. Feeding is a non-optional activity; if EBFoD is the most beneficial feeding option, each of these views will endorse an obligation to breastfeed where this is not too costly. Since each argument assumes you have an obligation to do what is best for your child within a particular set of choices, we will refer to ‘choice-specific’ duties and arguments.

Since choice-specific arguments are less demanding than the Maximal Benefit view Woollard considers, they are less vulnerable to Woollard’s costliness objections. Of course, one might still insist that choice-specific duties are too demanding. We will, however, suggest an alternative response.

An ongoing philosophical debate concerns the standards of parenting that ground the ‘right to rear’ one’s children. As Shields (2016) outlines, this debate has generated three broad views. The ‘Best Custodian’ (or ‘Best Available Parent’) view (Brighouse (1998: 737); Arneson (2000); Vallentyne (2003: 998); Dwyer (2011: Chapter 5); Gheaus (2021)) says parents have a right to rear only if they would provide as good a level of care, focusing solely on the child’s interests, as any other available carer. The ‘Abuse and Neglect’ view (Schoeman 1980: 17) sets a much lower bar, insisting that parental rights are retained in the absence of abuse and neglect. Finally, a range of ‘Dual-Interest’ views (Clayton (2006); Brighouse and Swift (2006); Shields (2016,2022)) accommodate both parents’ and children’s interests.

This debate analyses parents’ rights to retain decision-making authority (‘custody’) over their child’s life; it is thus concerned with particular kinds of state intervention, ultimately backed by enforcement through state institutions such as the police, courts, and social services. Health recommendations do not fall into this category. Still, one might ask equivalent questions, and come up with equivalent proposals, about the defeasible obligations parents have towards their children, where at least some of these obligations are *not* enforceable by the state. Thus, one might think parents are obliged to provide their children with at least as good an upbringing as any realistic alternative, but that failure to do so does not (always) imply loss of parental rights. This is still importantly different to, and less demanding than, the ‘Maximal Benefit’ view that Woollard (2018a) considers. While the Best Custodian approach demands that parents do at least as well as any realistic alternative guardian, the Maximal Benefit view demands that parents benefit their children as much as possible even if this is far more than any alternative arrangement would provide.

Alternatively, one might think parents only have obligations not to Abuse and Neglect their children. This view seems aligned with the harm-based argument Woollard considers in (2018b), though clearly one might hold an Abuse and Neglect view but reject the claim that not EBFoD constitutes abuse or neglect. Indeed, an Abuse and Neglect view might offer a minimal standard that supplements other views. For instance, one might think that parents should do at least as well as the best available alternative caregiver, but that if all available caregivers would be abusive or neglectful there is an obligation to do better than that (Gheaus 2021). Finally, one might hold (as Woollard seems to) a Dual-Interest view, where parents have obligations to benefit their children (some not enforceable by the state), but also entitlements to consider their own interests (Callahan 2019b).

As noted, Woollard (2018b) carefully elucidates the problems with framing feeding options other than EBFoD as harmful. We therefore focus on the other two positions. In Section 3.1. we assume for the sake of argument that EBFoD is always best for children and show that it is nonetheless optional on a Dual-Interest view. We then argue, in Section 3.2., that a Best Custodian view cannot support a duty to EBFoD.

A brief point before progressing. We acknowledge that the motivation for adopting a Best Custodian approach, in particular, is clearer when addressing the right to rear, since an infant being given to an alternative caregiver is precisely what’s at stake. Our interest in the Best Custodian view is as an approach that is infant-focused, but non-maximising. As Aksel Sterri has pointed out to us (personal communication), there are other possible views of parental obligation like this. For instance, a ‘Reasonable Custodian’ standard, where parental obligation is fixed not by actual alternative caregivers, but by some hypothetical reasonable caregiver. Alternatively, one might adopt a ‘Sufficient Benefit’ approach, whereby parents must meet some non-maximal standard of benefit.

However, we think that the standards offered by such alternatives are less clear than those suggested by a Best Custodian view. For this reason, and since the Best Custodian view is a mainstream option in the parental obligation literature, we focus on the Best Custodian view as representative of infant-focused but non-maximising approaches; but many arguments we offer against a Best Custodian view grounding the obligation to EBFoD also apply to such alternatives.

### Dual-Interest

Dual-Interest views fit the model outlined by choice-specific arguments straightforwardly. As outlined at the start of Section 3, choice-specific arguments say that for certain choices, parents must maximally benefit their children unless doing so would involve excessive costs. Dual-Interest views see parental obligations as constrained by parental interests; where benefitting your child would undermine your interests, you are sometimes permitted to refuse. A choice-specific version of this view simply restricts it to certain activities.

Infant feeding decisions are taken seriously because of their effect on infants’ physical health, which is doubly significant. First, infants are totally dependent on others. This dependency isn’t unique, but it is *universally* true of infants. Second, given their developmental stage, infants’ physical health often has long-term ramifications (Black et al. 2008).

Dual-Interest views acknowledge that, important as children’s interests are, they are not the only interests relevant to parenting decisions. Consider the fact that EBFoD requires breastfeeding-eligible parents to be available whenever an infant appears to be hungry and thus, it seems, to be nearly continually available to breastfeed. Such demandingness has consequences for parents’ interests: many activities that promote parents’ well-being are inconsistent with the demanding nature of EBFoD, and so foregoing them will be costs of EBFoD.

Breastfeeding is often difficult, and can cause painful, burdensome medical issues. An estimated 20% of breastfeeding parents suffer from lactation mastitis, “an acute, debilitating condition” (Cullinane et al. 2015) that causes pain in the breast(s), and sometimes fever. Symptoms include “a swollen area on your breast that may feel hot and painful to touch (…); a burning pain in your breast that might be constant or only when you breastfeed; nipple discharge, which may be white or contain streaks of blood; You may also get flu-like symptoms, such as aches, a high temperature, chills and tiredness.” (NHS 2022b) Typical treatment includes prescription antibiotics, which, though not harmful to babies “might make them irritable and restless.” (NHS 2022b) So, in just one common example, breastfeeding may cause a painful infection that will make feeding difficult and painful, and, if treated, may make the infants difficult to care for, notwithstanding other caring difficulties that might follow from issues in breastmilk supply.^3^

Where children are raised by several individuals, EBFoD’s implied division of labour raises interpersonal issues. Since EBFoD suggests that breastfeeding parents, primarily cis mothers, be free to feed a child in a near-unlimited capacity, this uneven distribution of labour may perpetuate ongoing tensions affecting all members of a household. Per Callahan (2019b), “[b]reastfeeding…constitutes a large portion of parental responsibilities in the early weeks and months of a baby’s life…breastfeeding may inculcate patterns of behaviour in which mothers assume parental responsibilities by default.”

There are two ways of understanding this point. The first—which we assume is Callahan’s—is as an external constraint on theories of parental obligation. Any realistic theory of obligation about a particular form of social life must have something to say about how obligations in that context fit into broader obligations. Even advocates of a maximal duty to benefit children will not think parents should take just *any* route to benefit children; they should not kill others to ensure their own child gets ahead, for instance. Viewed this way, Callahan’s observation is about the degree to which having EBFoD as a default assumption can contribute to broader social injustice by further cementing inequality in the distribution of labour between co-parents.

But Callahan’s point is also relevant to parents’ personal and political priorities. Inequality in expected parental obligation or labour caused by EBFoD harms breastfeeding-eligible parents, predominantly cis women, by reducing the extent to which they are able to engage in personal projects; insofar as they are committed to political liberation projects which depend on minimising the significance of biological difference in various kinds of work, it also harms them in a distinctive way by forcing them to abandon or compromise central values.

There are also costs to other family-members. EBFoD distributes important positive aspects of infant feeding, which, as noted, takes up a significant percentage of early care. Placing all (or most) of this opportunity with one parent can hinder non-feeding parents in feeling sufficiently involved in the process, and might impact parent–child bonding.^4^ Sharing feeding responsibilities equally (as EFF easily facilitates) is one way of mitigating against expertise and intimacy gaps established by hospital policies (where birthing parents typically spend the majority of the first days as lone carer) and parental leave policies (which allot significantly longer periods of leave to birthing parents). Given the demandingness of EBFoD, it will also negatively impact a breastfeeding parent’s ability to meet care obligations to other dependent adults in the family (we address other children in the Section 3.2.).

The pervasive requirements of EBFoD mean it impacts most other valuable activities in which breastfeeding-eligible parents could engage. In facilitating more even distributions between breastfeeding-eligible parents and others in their network, approaches to feeding which include formula feeding, including Exclusive Formula Feeding (EFF), facilitate greater participation in activities both of independent value and which contribute to the optimal functioning of a family or individual’s broader social network.

Thus, the costs involved in EBFoD often meet the ‘unless’ conditional contained within various choice-specific arguments: choosing to EBFoD will often involve slight or moderate costs, and may involve significant costs. However, it is worth noting that our discussion in this section considers not only benefits to breastfeeding-eligible parents but also to other adults in the family. Thus, choice-specific arguments as we originally framed them may be too narrow: it is not only costs to *you* that can affect your obligation to choose an option that is more beneficial to others, but also the costs to relevant others.

However, we also suggest that the view outlined here—based on a Dual-Interest approach to parental obligation—provides some grounds to push back against choice-specific arguments. The costs we have considered (and these constitute only a sample) *might* be seen to function as excuses, and thus fit into the terms of the cost-specific argument. But there is another way to see them; rather than taking breastfeeding as a default, and then seeing the various considerations as ‘excuses’, an alternative way of framing the Dual-Interest approach is to see costs and benefits to both the infant and adults of a family as ‘on all fours’ with each other. On a Dual-Interest approach, then, parental decisions should engage in trade-offs of interests (recognising that the interests of one family member may not be fully separable from the interests of others), aiming at what is best *for the family*. Costs to parents are not excuses, but rather competing considerations.

The costs and benefits we have considered are likely best judged by the breastfeeding-eligible parent, in tandem with other caregiving family-members. While the set of feeding options for young infants is limited—EBFoD; EFF; hybrid approaches—there is a significant range of ways in which this decision can impact and shape family life, and the options of various adults in the family. Thus, even if there sometimes *is* a duty to breastfeed (because the costs to adults in the family would not outweigh the benefits to the infant), it is not easily judged in particular instances by those unfamiliar with a particular family’s situation, including many healthcare professionals. And it is certainly not sufficiently rare to justify making national or international health guidance that pushes EBFoD as an obviously superior option. We suggest that this also makes it inappropriate for a liberal state to promote EBFoD over other viable feeding choices. This is for two reasons. First, even if parents had an obligation to breastfeed, further argument would be needed to support the claim that this obligation is such that the state should incentivise it (e.g., through public health promotion, financial subsidy at the cost of supporting other feeding options, or through greater institutional support), since not all parental obligations are legitimately enforceable by the state. Second, however, if we are successful in showing that there is no general obligation to breastfeed, this further strengthens the case for the state supporting a range of feeding options.

To be clear, this does not mean that the state should not provide support for breastfeeding through education, institutional support, and social policies that make it easier for parents to choose breastfeeding as a form of feeding.^5^ What it means is that the state should also, and equally, support parents who choose other forms of feeding.

### Best Custodian

Section 3.1 outlined how Dual-Interest approaches to parental obligation might challenge a universal duty to EBFoD. This section considers the ‘Best Custodian’ view. It may superficially seem that a Best Custodian view could not reject a duty to EBFoD, since Best Custodian views obligate parents to benefit children at least as much as other potential caregivers. But this is true only if we focus narrowly and implausibly on biomedical benefits to a single child, and perhaps not even then.

Best Custodian views can fit into a choice-specific framework by assuming that the Best Custodian standard applies only within certain kinds of choices. For instance, one might adopt a Best Custodian view that applies only to non-optional activities. Best Custodian views do imply one important constraint on choice-specific arguments. The logic inherent within Best Custodian views implies that the cost threshold is determined not externally or intuitively, but by virtue of what other available caregivers would do. With that clarified, we move to the question of whether a Best Custodian view could support an obligation to EBFoD.

It is necessary to highlight the difficulty in obtaining definitive information about purported medical benefits of breastfeeding and formula feeding. The singular message from health bodies (such as the WHO, CDC, NHS and HSE) is that breastfeeding offers numerous medical benefits to (at least) the health of the fed infant(s) (see Jung 2015: 72). The overwhelming issue with this is the lack of relevant randomly controlled trials (RCTs). The ethical objections to randomly imposing a feeding type on a new parent and child are obvious.^6^ Thus, most trials supporting common wisdom rely on observational studies.^7^ However, at least in economically developed countries, breastfeeding is strongly correlated with numerous other factors that impact child health outcomes, such as parental education level, wealth, and being in a non-smoking home. RCTs would allow researchers to control for such confounding factors, and with a large enough sample size, multivariate statistical analysis can isolate specifics among large clusters of correlated properties. Publication bias, conflations of exclusive and non-exclusive breastfeeding and variation of breastfeeding duration are also issues for much of the literature (see Jung (2015) for a critical summary). One meta-analysis had the following cautionary conclusion:

Because almost all the data in this review were gathered from observational studies, one should not infer causality based on these findings. Also, there is a wide range of quality of the body of evidence across different health outcomes. For future studies, clear subject selection criteria and definition of "exclusive breastfeeding," reliable collection of feeding data, controlling for important confounders including child-specific factors, and blinded assessment of the outcome measures will help. (Ip et al. 2007)

If the putative obligation to breastfeed rests on the claim that breastfeeding is the clear best option for an infant’s health, then this evidence base at least raises questions about such support. This raises a further epistemic and political issue—given that public health institutions and guidance are parental primary sources for most new families, the questionable evidence base coupled with a heavy-handed rhetorical framing risks making genuinely informed choice difficult and potentially constitutes a misapplication of political and institutional authority. However, while these issues are important to note, our aim in this paper is to show that *even if* the evidence was sound, there is no general obligation to breastfeed.

Returning to the view that a ‘Best Custodian’ perspective can also justify choosing not to breastfeed, three types of consideration support our claim.

The first is specific to the logic of the Best Custodian view, and thus does not apply to other child-focused but non-maximising approaches: if no available alternative caregiver would EBFoD one’s child, then on a Best Custodian view there is no obligation to do so; thus, on this view whether parents have an obligation to EBFoD depends on realistic alternative caregivers, and so there can be no universal obligation to EBFoD. Moreover, given the nature of breastfeeding, it will often be the case that no such alternative caregiver is available. Though it is possible to breastfeed an adopted/fostered baby (even if a breastfeeding-eligible parent has never been pregnant), the preparation is onerous and the amount of milk that can be produced varies considerably.^8^ Combined with the demandingness of EBFoD, this makes the likelihood of an alternative caregiver satisfying the recommended approach slim. Similarly, donor breast milk is now more widely available, but as discussed in the introduction, it still violates the no bottles requirement and sits lower in the WHO’s hierarchy of infant food sources. Therefore, on the Best Custodian view there will *rarely* be an obligation to breastfeed.

However, even if alternative caregivers would EBFoD, there are reasons to deny that a Best Custodian approach grounds an obligation to EBFoD. For many parents, the child whose feeding is under consideration is not their only child (Callahan 2019b: 214). Those who advance a Best Custodian view have two options concerning parents with multiple children. They might insist that parents have an obligation to do as well as alternative caregivers for each child, even when they cannot simultaneously do so for all their children. This implies that parents in such situations inevitably fail their obligations. Alternatively, a Best Custodian approach might say that parents should do at least as well as alternative caregivers for all of their children taken together. Thus, even if feeding decisions for an infant need not consider parents’ or other adults’ interests, they *do* need to consider other children in the family.

We have already mentioned the apparent need of breastfeeding parents to be *always available* to breastfeed a child according to EBFoD. Such guidance ignores the possibility that families have multiple children who could be breastfed. In such a situation, it is impossible for a parent to be always available to breastfeed *a particular* child, even if they are always available to breastfeed their children. While it is possible to breastfeed two children simultaneously, and more sequentially, the logistics coupled with the demandingness of the *always available* constraint make the recommendations very difficult to satisfy in practice.

More than this, however, children make demands on parental time in ways that do not involve breastfeeding, but which are equally important. If, per WHO recommendations, a breastfeeding-eligible parent is available to breastfeed until their first child is two, we might imagine some shock to the emotional system of this child when a new baby arrives with an entitlement to six months of EBFoD. In multi-parent families, the breastfeeding parent may make themselves always available to the new child as EBFoD suggests, while the other(s) assume near full responsibilities for the existing child(ren). This may prove a significant destabilising change for older children used to perpetual availability of a specific parent. For every new child who benefits from the demandingness of EBFoD, there is a related disruption and potential cost to previous children. We do not suggest that such decisions are easy, but merely that the demandingness of EBFoD on other dependency relations is rarely discussed in public health advocacy.

The foregoing argument shows why, even from a Best Custodian perspective, the choice to solely breastfeed an infant will not always be best for one’s children, and thus not always fulfil choice-specific duties to them. This shows that there is no clear Best-Custodian argument for an obligation to EBFoD for families with more than one child. We also think this is true for families with only one child. On a Best Custodian view, families with only one child must see the interests of adults in the family as subordinate to the interests of the child at least to the extent that any other realistic caregiver would. Defenders of ‘breast is best’ rhetoric might therefore think that cases such as these are the most straightforward cases of a duty to EBFoD.

We can demonstrate the problem with this assumption by considering a similar argument for older children. Consider a common parenting choice: whether and when to let non-infant children eat junk food. Focusing only on the effects of diet on the developing body and brain, the right decision might be to *never* accede. But there are benefits to allowing children to eat junk food, such that the decision about what is ‘best’ isn’t straightforward. Junk food is pleasurable in itself, and children who are barred from eating it may suffer social consequences, missing out on collective experiences with friends or being ostracised. We don’t suggest that parents should clearly allow children to eat junk; rather, our point is that other factors than biomedical health matter in developmental decision-making.

Infants’ constrained set of capacities means that the range of ways they can be harmed and benefitted is narrower: infants don’t suffer direct social stigma for feeding choices, for instance. Thus, biomedical health is a more prominent contributor to well-being. Moreover, as noted, the developmental stage of infancy means negative effects on biomedical health can have far-reaching consequences. But even for infants, biomedical effects do not exhaust harms and benefits. Assume that EBFoD really is biomedically best. There are also benefits to infants from a mixed or EFF approach. For instance, a parent who engages in EBFoD will find that doing so can “outstrip the responsibilities even of being a consistent caregiver with whom babies can form a secure attachment” (Callahan 2019b: 214). Thus, focusing on the biomedical benefits of breastfeeding may involve relative neglect of other benefits. Moreover, if feeding is important for bonding (as is frequently advised by breastfeeding advocates), it is noteworthy that children will benefit from bonds established with more than one care-giver, as EFF easily facilitates.^9^ Though it is possible to share feeding responsibilities in some breastfeeding arrangements, this requires the lactating parent to do the expressing necessary to furnish the other person with the breastmilk for the task.^10^ This supports our next point.

As discussed, the demanding nature of EBFoD is such that the division of labour in circumstances where a child is cared for by multiple adults (including family and professional caregivers) is stark. This way of feeding precludes ongoing participation in many other activities—be they professional, leisurely, or educational—that are inconsistent with being perpetually available for feeding. This division of labour may suit many parents, but not all. The physically and psychologically demanding nature of early parenting may mean that a breastfeeding parent feels unfairly put-upon by the extent of the role, which may produce friction and a sense of inequality in the family. The atmosphere of a home is important to the wellbeing and development of a child (Heinonen et al. 2018; von Bonsdorff et al. 2019). Parents’ welfare isn’t easily separated from children’s welfare; if trying to EBFoD despite pain, unhappiness and disruption means parents have lower well-being, this will likely harm the child. Additionally, EBFoD’s demandingness means its consistency with paid work varies with available support. In economically developed countries, breastfeeding covaries with both social class and parental education (Heck et al. 2006; HSE 2016; UNICEF UK 2017). Thus, EFF may benefit infants by facilitating bonding with multiple caregivers, and the fairer distribution of responsibilities and opportunities EFF permits can contribute to a happier and more stable household environment.

Though the factors that go into deciding what is best are more limited on a Best Custodian view—since they relate only to children and not to adults in the family—the range is still considerable, and it will be difficult for anyone not well-acquainted with individuals’ circumstances to decide which option is best.

Thus, we have shown that even from a narrow perspective, the claim that breastfeeding-eligible parents must EBFoD is not plausible. Even if we accept a choice-specific argument, it doesn’t support a general duty to EBFoD.^11^

## Pragmatic Satisficing

Section 3 outlined several ‘choice-specific’ arguments for an obligation to EBFoD that are less demanding than Woollard’s main target. We examined why, even if choice-specific arguments are correct, they don’t establish an obligation to breastfeed: on any reasonable view of parental obligation, breast isn’t always best. Further, even though there are some cases where EBFoD *is* the best choice, the intimacy of the relevant factors makes it difficult for outsiders to make this judgement, and thus inappropriate to blame mothers who do not breastfeed, or to promote breastfeeding as a default, or as obligatory. As we have suggested, this makes it unlikely to be an appropriate use of state resources to exclusively or selectively support EBFoD over other feeding options.

However, one issue remains. Pressure to breastfeed also comes from within. Parents—given the dominant cultural framing of parenthood, especially cis mothers—are, as Woollard notes, encouraged to *see themselves* as having demanding obligations. This may lead many to feel that they fail their children when they do not make the ‘right’ choice.

Whereas Woollard’s argument criticises a duty to maximally benefit one’s children, our argument has noted that what is best is a complex question, and typically difficult for those outside a family to judge accurately. But this still leaves open the possibility that for some parents, EBFoD *is* best when all relevant factors are considered, and is therefore obligatory. We may not know of any particular family that they are in this situation. But, at least on the basis of our argument thus far, one might think parents will know which situation they are in. Thus, parents who could breastfeed but do not do so, and who are in a situation where that would be best, *should feel guilty.* Indeed, our argument raises the prospect of a further, parallel issue: parents who do EBFoD, but for whom it is in fact not best (all things considered) also do the wrong thing.

We have two responses. On a Dual-Interest view, some ways in which a particular feeding choice may be overall best concern permissible but non-obligatory benefits to one or more adult family-members. Thus, parents may permissibly either do what is overall best, or what is best for their child, declining permissible benefits for themselves.

More generally, all we have said is that parents are *best* placed to judge what is overall best, not that they are infallible. Sometimes it may be clear what is best; but sometimes it won’t be. And sometimes the best thing is simply to choose what is *good enough*, i.e., to ‘satisfice’. ‘True’ satisficing—choosing a suboptimal option, even when the better option would involve no additional cost—is controversial. But it is uncontroversially rational to sometimes employ ‘pragmatic’ satisficing as a ‘stopping rule’ (e.g., Schmidtz (2010)). Stopping rules involve setting ‘good enough’ thresholds, and taking options that clear those thresholds, even though better options may be available. This can be rational for two reasons. First, the range of options might be large. Imagine that you are choosing where to go on holiday. The range of possible destinations that meet certain criteria (budget, location, weather) is large. You research several options, finding one that seems appealing. If you kept looking, you might find something better; but to carry on looking requires time and effort that *might* not be outweighed by the additional benefit.

This is the typical case of pragmatic satisficing (Byron 2010). Now, imagine that you are choosing a holiday, and only a few destinations are affordable. Thus, you can thoroughly review all available options. However, you have several criteria for a holiday: weather, food, location, etc. Each destination does a bit better than competitors on some variables, and a bit worse on others. You may be unsure what the best option is. Does slightly better food make up for less predictable weather? By how much? It may not be obvious. We do not know ourselves perfectly, and once companions are added to the mix things become even more complicated. And so it seems reasonable to just pick one of the attractive destinations. All of them will make for a good holiday!

Infant-feeding raises particular complexities for epistemic confidence. A flexible trial-and-error strategy is limited (perhaps precluded) by the nature of milk supply and sensitive infant stomachs. One doesn’t know what condition one will be in when a baby arrives, and understanding how EBFoD, EFF or the intermediary approaches will mix with various complex strands of family life and health would require incredible self-knowledge and knowledge of others. For most, the decision requires some trust. Thus, for many families it will be unclear which feeding option is best, because each has different costs and benefits which are difficult to balance. In such cases, it is reasonable to engage in pragmatic satisficing: pick a feeding option that looks like it will work for everyone involved, without confidence that it is best. Thus, at least in such situations, someone’s not choosing the best feeding option—for their child or for their whole family—does not require them to feel guilty, since it is no failure of an obligation.

## Conclusion

Whereas existing work on putative obligations to breastfeed, primarily from Woollard, has focused either on a very demanding account of parental obligation (Maximal Benefit) or a very minimal account (avoiding significant harm), we have considered two more moderate accounts of parental obligation, drawing on broader ‘right to rear’ literature. We have shown that on a Dual-Interest approach there can be no obligation to breastfeed because of the significant interests of adults—including but not limited to the breastfeeding-eligible parent—that may be promoted by other forms of feeding, including EFF. We then showed, more surprisingly, that no obligation to EBFoD can be derived from a more demanding view of parental obligation (Best Custodian) because existing justifications rely too narrowly on biomedical benefit. Finally, we defended the view that this decision is best left to parents and is sufficiently complex to warrant a pragmatic satisficing approach. In short, parents should be left to make the feeding decisions they view as likely to be sufficiently good for their families. Most other individuals should not try to persuade them to act otherwise, while medical and other institutions should acknowledge that feeding decisions are complex, and that each option has benefits and downsides. Moreover, the state should not exercise its power to exclusively or selectively promote and support EBFoD over a range of other reasonable feeding options. As a result, all parents should be supported within a range of reasonable feeding decisions, which includes exclusive formula feeding.

## Data Availability

Not Applicable.

## Footnotes

1 Kukla (2006: 159–60) (writing as Rebecca Kukla) describes the case in the USA: “Contemporary breastfeeding advocacy materials… form an excellent example of an intervention aimed directly at mothers’ choices and behaviours and premised on mothers’ special responsibilities for a certain domain of healthcare.”

2 See e.g., NHS (2022a) and HSE ([Bibr CR20][Bibr CR21]) guidance. A moralistic streak pervades the advice: though no strong evidence links modest alcohol consumption and negative foetal/maternal outcomes, it is prohibited; despite evidence of harm for some cleaning products (Cordier et al. 2012)), cleaning caution appears on neither list.

3 See Kukla (2006:163–70) for the complex social, sexual and cultural challenges breastfeeding can pose and the contrast between this and the advocacy literature focusing on “experiences of joy, fulfilment, pleasure and maternal bliss, pausing only occasionally to concede the rare mechanical difficulty” (Kukla 2006:168).

4 Hairston et al. (2019) conclude that it is feeding *per se* and not feeding method that impacts bonding, though we acknowledge the small sample size and observational nature of the study.

5 The authors agree that it is important that families should be given very robust support in feeding their children, be it via EBFoD, mixed feeding or EFF. However, dogmatic institutional rhetoric in favour of EBFoD and against EFF should not be confused with actual material support of EBFoD or mixed feeding.

6 Though, one might argue that the institutional pressure to breastfeed, coupled with economic sanctions on formula and routine exaggeration of the health benefits of breastfeeding, come very close to making it difficult to make an informed choice not to breastfeed, even if most agree it would be wrong to impose EFF on families who might otherwise breastfeed.

7 This feature of the history of breastfeeding literature is given as a motivation for the PROBIT study (Kramer et al. 2001), which avoids numerous issues of observational studies by using contrasts of large populations of Belarussian maternity hospitals (some of which randomly received breastfeeding promotion interventions and some of which didn’t). The only benefits supported by the study were a decreased risk of gastrointestinal tract infections (9.1% vs 13.2%) and atopic eczema (3.3% vs 6.3%). See Kramer (2009) and Martens (2012) for discussion of limitations.

8 La Leche League International (2018).

9 Writing in a blog for LLLI, Schnell (2020) advises, non-breastfeeding parents to pursue other means of bonding, including options where we can find no suggestion that these activities are established as supportive of bonding, such as nappy-changing and bathing. In the examples with a better-established claim of bonding support (“baby-wearing” and play), it is made clear that these should not interfere with the breastfeeding “dyad”.

10 Additionally, this is expressly ruled out in the WHO recommendations.

11 Much of our argument in this section also provides reason to doubt that a Maximal Benefit view supports an obligation to EBFoD.
